# High-Throughput Sequencing of a South American Amerindian

**DOI:** 10.1371/journal.pone.0083340

**Published:** 2013-12-30

**Authors:** André M. Ribeiro-dos-Santos, Jorge Estefano Santana de Souza, Renan Almeida, Dayse O. Alencar, Maria Silvanira Barbosa, Leonor Gusmão, Wilson A. Silva, Sandro J. de Souza, Artur Silva, Ândrea Ribeiro-dos-Santos, Sylvain Darnet, Sidney Santos

**Affiliations:** 1 Instituto de Ciências Biológicas, Universidade Federal do Pará, Belém, Pará, Brazil; 2 Centro Regional de Hemoterapia, Faculdade de Medicina de Ribeirão Preto, Universidade de São Paulo, Ribeirão Preto, São Paulo, Brazil; 3 Institute of Bioinformatics and Biotechnology, São Paulo, São Paulo, Brazil; 4 Institute of Molecular Pathology and Immunology, University of Porto, Porto, Portugal; 5 Departamento de Genética, Faculdade de Medicina de Ribeirão Preto, Universidade de São Paulo, Ribeirão Preto, São Paulo, Brazil; 6 Brain Institute, Universidade Federal do Rio Grande do Norte, Natal, Rio Grande do Norte, Brazil; Universitat Pompeu Fabra, Spain

## Abstract

The emergence of next-generation sequencing technologies allowed access to the vast amounts of information that are contained in the human genome. This information has contributed to the understanding of individual and population-based variability and improved the understanding of the evolutionary history of different human groups. However, the genome of a representative of the Amerindian populations had not been previously sequenced. Thus, the genome of an individual from a South American tribe was completely sequenced to further the understanding of the genetic variability of Amerindians. A total of 36.8 giga base pairs (Gbp) were sequenced and aligned with the human genome. These Gbp corresponded to 95.92% of the human genome with an estimated miscall rate of 0.0035 per sequenced bp. The data obtained from the alignment were used for SNP (single-nucleotide) and INDEL (insertion-deletion) calling, which resulted in the identification of 502,017 polymorphisms, of which 32,275 were potentially new high-confidence SNPs and 33,795 new INDELs, specific of South Native American populations. The authenticity of the sample as a member of the South Native American populations was confirmed through the analysis of the uniparental (maternal and paternal) lineages. The autosomal comparison distinguished the investigated sample from others continental populations and revealed a close relation to the Eastern Asian populations and Aboriginal Australian. Although, the findings did not discard the classical model of America settlement; it brought new insides to the understanding of the human population history. The present study indicates a remarkable genetic variability in human populations that must still be identified and contributes to the understanding of the genetic variability of South Native American populations and of the human populations history.

## Introduction

The emergence of next-generation sequencing (NGS) technologies, such as Solexa (Illumina) [1], 454 (Roche) [2] and SOLiD (Life Technologies) [3], allowed access to the vast amounts of information that are contained in the genomes of various organisms. In recent years, two large projects, HapMap [4] and the 1,000 Genomes Project [5], and other more specific initiatives [6]–[14] sought to document the genetic variability in the human genomes of different ethnic and geographic groups. This type of investigation allowed the identification of rare genetic variants [11], [15], the ability to make more precise inferences from association studies with complex diseases [16], and the formulation of new insights in the history of the formation and migration of human populations [6]–[10],[12]–[14],[17]–[19].

The Americas were the last continents to be occupied by human populations. According to the most widely accepted model, which is based on anthropological, archaeological, and genetic evidence [18], [20]–[22], the American natives originated in Eastern Asia 20 to 30 thousand years ago (kya) and expanded across the Americas along the North-South direction [18]. Despite the large number of publications on the occupation of the Americas [18], [20], [23], [24], several questions have not yet been answered. Information on individual and/or population-based genetic variability, such as the information provided by whole genome sequencing (WGS), might contribute to the solution of many of these questions.

The literature records at least 15 fully annotated WGSs of individuals from European [8], Asian [6], [7], [11], [13]–[15], Oceanic [12], and African [9] populations, but have no Native American representatives. To obtain a better understanding of the genetic variability of Native Americans and to infer genetic associations with complex diseases, such as diabetes mellitus type 2 and coronary artery diseases frequently detected in South American populations, the full genome of an individual from a South American indigenous population was sequenced. The data obtained served as a basis for the comparison with representatives from different geographic areas, the discovery of new polymorphisms (SNPs – single nucleotide polymorphisms; and INDEL - insertion/deletion polymorphisms) and represents the first WGS of a South Native American individual.

## Results

### Whole genome sequencing and mapping

The two *mate-paired* full-slide runs that were performed produced 36.8 Gbp of raw data (Table 1). The total number of Gbp that were mapped on the human genome was 31.4 (85.5%). Of these mapped Gbp, 25.9 Gbp (70.34%) exhibited single alignment, and 35.62% of the reads were paired. A 95.92% genome mapping was achieved with an average sequencing coverage of 8.23× and a physical coverage of 99.83×. The sequencing error rate was estimated to be 0.0035 per base pair (bp), as suggested by Fujimoto *et al.* [11].

**Table 1:** Sequencing runs and mapping of the Amerindian sample genome.

	Run	
	1st*	2nd**	Total
**Read Length**	50/50	50/50	
**Number of Reads**	95,242,345	640,615,685	735,858,030
**Total Bases (Gbp)**	4.8	32.0	36.8
**Mapped Reads (%)**	79.50%	86.44%	85.54%
**Unique Mapped Reads (%)**	60.04%	71.88%	70.34%
**Unmapped Reads (%)**	20.50%	13.56%	14.46%
**Mapped Pairs (%)**	68.63%	78.49%	77.21%
**Paired Reads (%)**	53.60%	32.95%	35.62%

*Physical coverage of 10.2× with a mean insertion size of 1238 bp;

**Physical coverage of 7.37× with a mean insertion size of 1,055 bp.

### Identification and annotation of SNPs and INDELs

Approximately 2.1 million SNP and INDEL polymorphisms were identified. Of these polymorphisms, 502,017 were considered to have high confidence (coverage equal to or higher than 10× and a minimum support of 4×), of which 435,947 (86.84%) were already described in the Single Nucleotide Polymorphism database (dbSNP) and 66,070 (32,280 SNPs and 33,785) were considered potentially new polymorphisms (Table 2).

**Table 2:** High-quality polymorphisms identified and not identified in the dbSNP build 135 database.

Mutation Type	Total	%*	Described in dbSNP	%*	Not described in dbSNP	%*
SNP	433,310	86.3%	401,035	92.0%	32,275	48.8%
Small INDEL	46,657	9.3%	34,736	8.0%	11,921	18.0%
Large INDEL	22,050	4.4%	176	0.0%	21,874	33.1%
**Total**	502,017	100.0%	435,947	86.8%	66,070	13.2%

*These percentages correspond to the column total.

The total number of high-confidence SNPs with coverage equal to or higher than 10× was 433,310 (available at Table S1). These polymorphisms were classified according to their mutation type (transition or transversion) and their relative position to the closest gene, 5′-UTR, introns, and other gene characteristics (e.g., synonymous, non-synonymous, stop codon loss, stop codon gain, intronic, 5′-UTR, 3′-UTR, near 3′ end of gene, 5′ splice site, and 3′ splice site; Table 3). The mutations were more frequently found at the intronic regions; the next most common locations were the 3′ end of the gene (500 bp after the 3′ end of the gene) and the 3′-UTR. Of the 32,275 potentially new SNPs, 1,899 (5.88%) and 123 (0.38%) exhibited a coverage that was equal to or higher than 20× and 50×, respectively. The following analyses were based on the complete set of high-quality SNPs, as described above.

**Table 3:** Nature (genomic position and type of mutation) of the SNPs identified in the dbSNP database.

Annotation	Identified SNPs	Unidentified SNPs	Total
Total	401,035	32,275	433,310
Transitions	272,498	24,462	296,960
Transversions	128,537	7,813	136,350
Synonymous	1,318	91	1,409
Non-synonymous	1,179	157	1,336
Stop codon loss	7	1	8
Stop codon gain	16	14	30
Intronic	36,949	3,041	39,990
5′-UTR	205	13	218
3′-UTR	2,166	152	2,318
Near 5′ end of gene	1,664	92	1,756
Near 3′ end of gene	2,303	173	2,476
5′ splice site	2,166	152	2,318
3′ splice site	3	3	6

### Maternal and paternal lineages

The maternal and paternal lineages of the sample were assessed through a comparison of the SNPs found in the mitochondrial DNA (mtDNA) and the SNPs found in chromosome Y (Y-DNA) [18], [25], [26]. The mtDNA exhibited an average coverage of 729× and 41 variable points were detected along the mitochondrial genome; among those 16 mutations (064 C>T; 146 T>C; 153 A>G; 235 A>G; 663 A>G; 1,736 A>G; 4,248 T>C; 4,824 A>G; 8,027 G>A; 8,794 C>T; 12,007 G>A; 16,111 C>T; 16,223 C>T; 16,290 C>T; 16,319 G>A; and 16,362 T>C) are commonly found among the Amerindian populations, and classified the sample within the A2 haplogroup [21], [25]–[30]. The analysis of the Y-DNA revealed the mutations M242, M346, and M3, which allowed the classification of the sample within haplogroup Q1a3a* [31].

### HapMap's population-based comparisons

We compared the set of high-confidence SNPs that were found in this study with those obtained in the HapMap project phases 1, 2, and 3 [4]. Of the reliable SNPs identified in the sample, 205,634 had not been previously genotyped in any of the populations included in the database, whereas 227,676 had already been found in at least one individual. The Asian populations (CHB and JPT) shared the most, 222,644 SNPs (Figure S1). Moreover, these comparisons revealed that 2,955 SNPs genotyped in all of the populations included at HapMap. The genotype data regarding these 2,955 SNPs (dataset A, see Material and Methods) was applied to following analysis.

**Figure 1:**
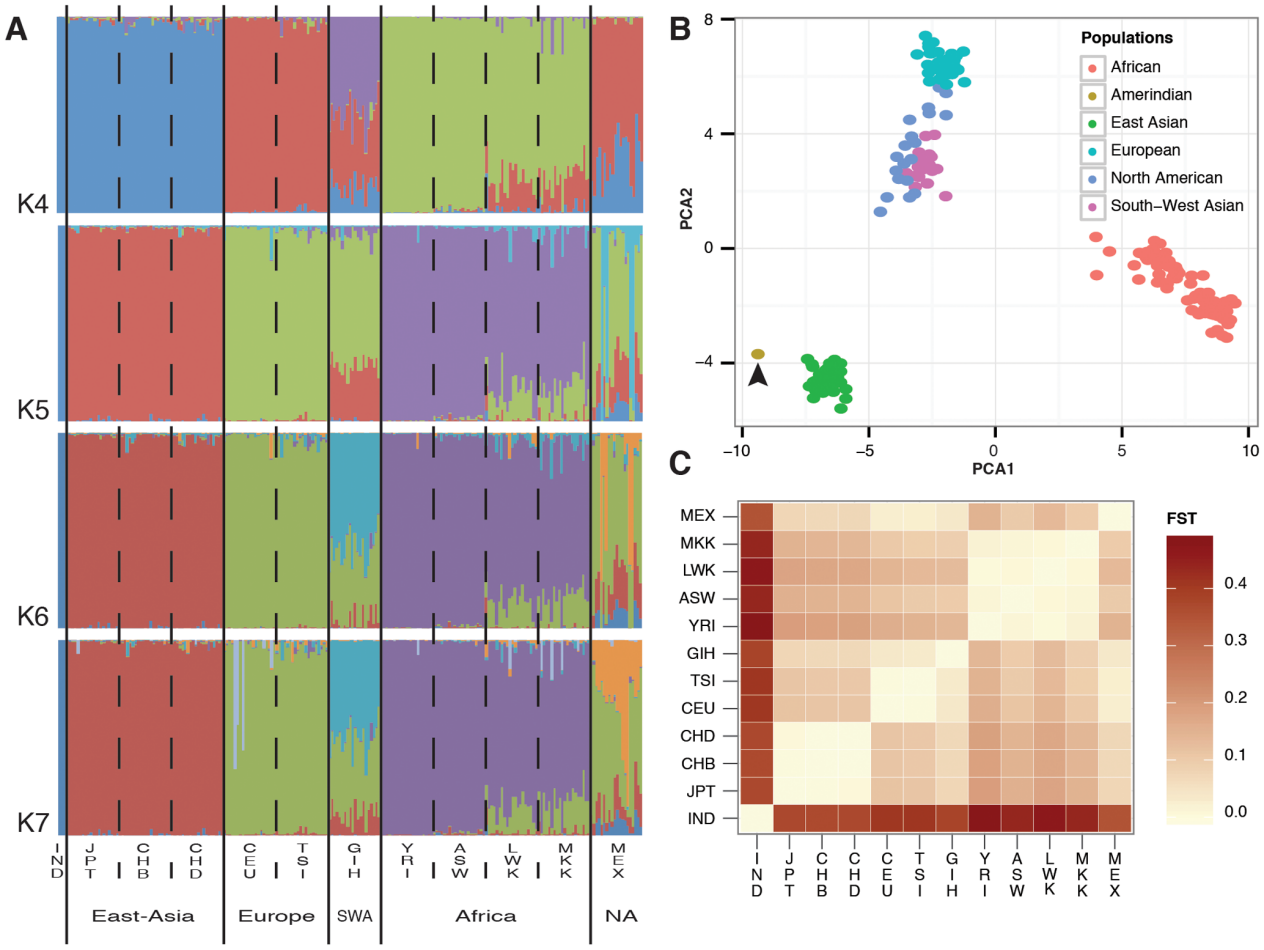
Comparative population-based genetic analysis of the Amerindian with the populations in the HapMap database. The Amerindian sample (IND) was compared to 20 randomly selected samples from the following populations in the HapMap database: JPT (Japanese in Tokyo, Japan), CHD (Chinese in metropolitan Denver, Colorado, USA), CHB (Han Chinese in Beijing, China), CEU (residents of Utah, Nevada, USA with Northern and Western European ancestry from the CEPH collection), TSI (Tuscans in Italy), GIH (Gujarati Indians in Houston, Texas, USA), YRI (Yoruba in Ibadan, Nigeria), ASW (individuals with African ancestry in Southwest USA), LWK (Luhya in Webuye, Kenya), MKK (Maasai in Kinyawa, Kenya), and MEX (individuals with Mexican ancestry in Los Angeles, California, USA). The populations were clustered according to their geographic origin as follows: East-Asia (JPT, CHB, and CHD), Europe (CEU and TSI), South-West Asia (SWA, formed by the GIH population), Africa (YRI, ASW, LWK, and MKK) and North America (NA, formed by the MEX population). A) Diagram of the genetic contribution of the models for a value of K in the range of four to seven. The x-axis represents the different samples that were clustered according to the population and the geographic area of origin. B) Principal Component Analysis (PCA) of the Amerindian sample (indicated with the arrow) and the samples extracted from the HapMap database. The abscissa represents the 1^st^ component, and the ordinate represents the 2^nd^ component. C) Heat map of the FST index between the investigated populations and the Amerindian sample.

The results of STRUCTURE analyses are represented at Figure 1A. With a K value of four, the Amerindian sample clustered with the Asian populations (JPT, CHD, and CHB), with only 1% of external contribution. The results also indicated a group of the European populations (CEU and TSI), a group of the African populations (YRI, ASW, LWK, and MKK), and the admixed group of Mexican (MEX) and Indian (GIH) samples.

With a K value in the range of five to seven, no gene flow with the Native American was found. At this level of analysis, the European (CEU and TSI), Asian (JPT, CHD and CHB), and African (YRI, ASW, LWK and MKK) populations were found to form homogeneous groups, although the LWK and MKK populations exhibited a small degree of mixture, mainly with Europeans. As expected, the Mexican (MEX) and Indian (GIH) populations exhibited a high proportion of admixture with the various populations.

Two other approaches were used to estimate the genetic differences: discriminant analysis of the principal components (DAPC), which illustrated the differences among the individuals and populations (Figure 1B); and the fixation index (FST) among the 12 populations. To facilitate the visualization of the results, the measures were plotted using a heat map (Figure 1C). Both results showed that the sample was isolated from the others populations. With the exception of the Mexican population, which is known to result from a mixture of Europeans and Amerindians, the Eastern Asian populations (JPT, CHB, and CHD) were found to be closest to the South Amerindian sample. The African and European populations were the most distant populations from the sample.

### 1,000 genomes population-based comparisons

The high-confidence SNPs were also compared with those found by 1,000 genomes project phase 1 [5] and the Aboriginal Australian [12]. It was found a set of 13,177 SNPs (dataset B, see Material and Methods) used for STRUCTURE and Threepop analyses. The analysis of f_3_ statistics did not reveal any gene flow involving the Native American sample with any other population (Table 4).

**Table 4:** Three-population admixture test *f_3_* *.

Outgroup	Ingroup 1	Ingroup 2	*f_3_*	SD	Z score
IND	ABO	ASN	0,066	0,003	22,001
IND	ABO	EUR	0,080	0,003	24,311
IND	ABO	AFR	0,092	0,004	24,460
IND	ABO	AMR	0,067	0,003	22,704
IND	ASN	EUR	0,165	0,004	37,771
IND	ASN	AFR	0,165	0,004	38,438
IND	ASN	AMR	0,158	0,004	37,665
IND	EUR	AFR	0,184	0,004	41,561
IND	EUR	AMR	0,168	0,004	38,888
IND	AFR	AMR	0,167	0,004	39,274

*The *f_3_* tests the hypothesis that the outgroup is result of admixture of two ingroups (see Material and Methods). The groups were identified as: IND - South Native American; ABO - Australian Aboriginal (Rasmussen et al. 2011); ASN - Asian populations (CHS, CHB and JPT); EUR - European populations (CEU, IBS, TSI, FIN and GBR); AFR - African populations (ASW, LWK, MKK and YRI); and AMR - American populations (CLM, PUR and MXL).

**Figure 2:**
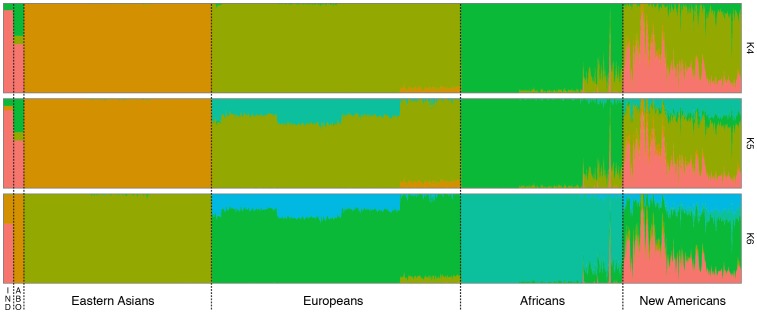
Population genetic structure analysis of 1,000 genomes project's, Aboriginal Australian and Native South American genotype dataset. The diagram of genetic contribution was obtained using Structure software for models of 4 to 6 subpopulations. The populations were grouped labeled as follow, according to their major continental ancestry: IND (Native South American individual, this work); ABO (Aboriginal Australian individual, Rasmussen et al. 2011); Eastern Asian (CHS, CHB and JPT); Europeans (CEU, IBS, TSI, FIN and GBR); African (ASW, LWK, MKK and YRI); and New Americans (CLM, PUR and MXL). The plot represents the rate of contribution from each subpopulation (colors) to the samples (x axis).

The STRUCTURE analyses (Figure 2, Figure S2) reveal an important contribution of Eastern Asian populations to the Native American sample (K less than 4). For K between 4 and 5, the sample distinguished from others groups, with more than 88% specific contribution. For K values higher or equal to 6, a new group emerges, isolating the Australian Aboriginal sample with more than 90% of specific contribution. Interesting to notice that for these analyses our sample presented 30% of contribution from the Aboriginal population.

The American populations (CLM, PUR and MXL) presented a similar scheme of contribution for K values higher or equals to 5. They had the same population-structure formed by three major groups: European, African and Native American.

## Discussion

The aim of the present study was to describe potential new genetic variations to be further analyzed among South Native American populations through the use of NGS technologies. The specialized literature describes the fully sequenced genomes of samples of other populations, including samples of Japanese [11], Chinese [15], Korean [6], [7], Indian [13], [14], Aboriginal Australian [12], and African [9] populations; these samples are available for comparison analyses. The accumulation of additional data might contribute to a better understanding of the genetic diversity that is present in those populations and to an improved historical description of the expansion of the human population across the various continents.

The results of the present study contribute to the catalogs of genetic variation that have been organized by various projects, such as the 1,000 Genome [5] and the HapMap [4] projects, which did not include any South Native American samples until the moment.

The comparison of the findings with the available dbSNP, HapMap and 1,000 genomes datasets showed a high number of potentially new polymorphisms. This result indicated the importance of acquiring information on the genetic variability of human populations through the sequencing of individuals from different ethnic groups, particularly those that have not been previously investigated, such as the Native Americans. This approach is specially important to the study of isolated populations regarding their healthy, such as, the finding of markers associated to complex diseases like diabetes type 2, coronary diseases, hypertension and others.

The analyses performed limit the possibility of sequencing errors and sample contamination. This study mapping, genome coverage and SNP detection was similar to previous studies [12], [13], [15]. The quality of these results is attested by the low error rate (estimated as 0.0035 per bp) as the one obtained by Fujimoto *et al.* [11].

A STRUCTURE analyses was performed to establish the relationship between the investigated sample and samples from several different populations. The high number of SNPs shared with Eastern Asian populations corroborate to the classical model of the America occupation.

The sample ancestry origin was through the analysis of the maternal (mitochondrial DNA) and paternal (Y-DNA) lineages. The A2 mitochondrial haplogroup is typical of the indigenous populations of the Americas, including the South American indigenous groups, and it is found in approximately 15% of the Amazonian tribes [32], 3.7% of the Andean tribes [33], and 5.9% of the southern South American tribes [33]. The Q1a3a* Y-DNA haplogroup [31] is specific to Native American tribes [34]. It is the most frequent among the South Native American tribes (more than 90%; [35]). With rare exceptions, other lineages belonging to clade Q were detected, although at a much lower frequency; (e.g. [36]–[41]). Therefore, the analysis of the uniparental markers indicated that both lineages were derived from South Native American populations.

The introgression of European genes into Native American populations, particularly through the crossings between European males and Amerindian females [42] is relatively well known in the specialized literature. The dataset A analyses presented no admixture of the sample with any continental population that comprised more than four subpopulations. The 1,000 genomes dataset analyses with K larger than 5 no contribution of African or European populations was found. To further exploit our data, for possible recent admixtures, it was applied the f_3_ statistic to the 1,000 genomes dataset and no admixture scheme was statistical significant. The data support the conclusion that the investigated sample does not exhibit traits of a recent interethnic mixture and, thus, can be considered an authentic South Native American individual.

Although, no evidence of recent admixture was found, the 1,000 genomes STRUCTURE analyses indicated an important steady contribution of the Aboriginal Australian to the South Native American sample was found for K larger than 5. Three scenarios can explain similarities between the Aboriginal and our sample: i) an ancient shared history between the groups [43]; ii) ancient migrations, others than the classical Bering model [35], [44]; and iii) due to derive considering each group consist of only one sample. This matter can only be clarified by the expansion of the number of samples and dataset available to analysis.

The present study identified 32,275 potentially new SNPs with a low error rate. These new SNPs may contribute to the understanding of the composition and genetic history of South Native American populations. The authenticity of the sample was demonstrated through the analysis of the maternal, paternal, and autosomal lineages. In addition, an autosomal analysis was able to distinguish the sample from the other continental populations and showed that it is close to the Eastern Asian populations. Other important finding was the indication of a shared history between our sample and the Australian Aboriginal, although no conclusion may be taken. Although, the findings did not discard the classical model of America settlement; it brought new insides to the understanding of the human population history.

## Materials and Methods

### Ethics Statement

Ethical consent was obtained according to the Helsinki Declaration. Ethical approval was obtained from the Brazilian National Committee on Research Ethics (CONEP- Parecer No 1062/2006). A signed informed consent was obtained from the village leaders since most subjects were not literate in Portuguese, but all subjects provided verbal assent to participate. A FUNAI/FUNASA health agent, who helped explain the aims and scope of the study to individuals, accompanied all activities.

### Preparation of the Sample and Sequencing Library

The genomic DNA was obtained from an Amazon tribe male healthy individual. Along the years, our group gathered a pool of samples from several Amazon tribes. To guarantee the best ethical behavior regarding our samples and their tribes, the studied sample was selected randomly from our pool of male samples together with Roewer's Amazon samples (recently investigated in Roewer *et al.* [35]). From the genomic DNA sample, a 1,000-bp insert mate-pair library was prepared. The library was sequenced using the SOLiD v.4 Plus platform (*Life Technologies, CA, USA*) according to the manufacturer's protocol. Based on the purpose of the present study, two runs were performed each one in a full slide.

### Ultra-Deep Sequencing of SOLiD v.4 Plus and Mapping

The SOLiD platform generated thousands of reads with a length of 50 bp. The results were transferred to the processing server, where both runs were aligned to the reference human genome (NCBI Genome Reference build 37 – HG19/GRCh37 Feb. 2009) using the Bioscope™ v.1.3 platform (*Life Technologies, CA, USA*) following the manufacturer's recommended protocol. After the alignment, the output of the Bioscope software was converted to the BAM format, and the sequencings were paired.

### Error Rate

The sequencing error rate was estimated using the data from chromosomes X and Y, as suggested by Fujimoto *et al.* [11]. The regions that were classified by Repeat Marker [45] to contain pseudo-autosomal contigs, short repeats, and repeat sequences were not considered. The predominant base call at each position was considered correct, and the other calls were considered erroneous.

### Polymorphism call

The polymorphisms were called using the mpileup software of the SAMtools v.0.1.17 package [46] and the Bioscope v.1.3 platform. The following criteria were applied for the detection of the high-quality SNPs: (1) the mapping quality of the reads (MAPQ) should be greater than 25 (Phred scale), (2) the SNP coverage should be equal to or greater than 10×, and (3) the 10-bp window can contain up to three polymorphisms. The polymorphism zygousis was determined according to the filtered total and variant coverage. A variant was called as heterozygous when the ratio of variant coverage (variant/total) was less than 0.66 and mutant homozygous when the ratio is above 0.66. The large INDEL calling analyzes the read pairs and identifies those that exhibit significant changes in the average distance between pairs; the results were sorted according to the following criteria: (1) minimum coverage of 3×, (2) pairing quality equal to or greater than 25 (Phred scale), (3) mapping with a minimum size of 30 bp, and (4) maximum coverage of 10,000×. The small INDEL calling analyzed the small gaps in the alignment of the reads, and the results were sorted according to the following criteria: (1) the polymorphism was supported by at least two pieces of evidence and (2) the quality of the best mapping among the reads should be equal to or greater than five. All of the detected polymorphisms were compared to the polymorphisms described in the dbSNP build 135 database [47] to identify the rsID (dbSNP identification code) and genomic loci.

### Maternal and Paternal linage analysis

The sample ancestry was inferred studying the maternal and paternal linage. Both linages were based on motifs (of mitochondrial and Y DNA, respectively) that characterize ethnic groups. The mitochondrial mutations were identified aligning the reads from the mitochondrial to the Andrews Reference Genome (rCRS, Revised Cambridge Reference Genome) [48], [49]. The haplogroup was determined according to the PhyloTree build 15 [26] classification and others papers [21], [25], [27]–[30]. The Y-DNA haplogroup was determined according to Karafet *et al.* [31] binary classification tree.

### Population-based Comparisons

To further investigate our sample, it was collect two dataset of bi-allelic SNPs genotype data. The first dataset (dataset A) consists in 220 HapMap samples' and our sample's genotype data for 2,955 SNPs genotyped for all HapMap's samples and identified as high quality in our sample. The 220 samples were selected randomly, including 20 representatives for each population: Japanese in Tokyo, Japan (JPT); Han Chinese in Beijing, China (CHB); Chinese in Metropolitan Denver, Colorado (CHD); Gujarati Indians in Houston, Texas (GIH); Yoruban in Ibadan, Nigeria (YRI); African ancestry in Southwest USA (ASW); Maasai in Kinyawa, Kenya (MKK); Luhya in Webuye (LWK); Utah residents with Northern and Western European ancestry from the CEPH collection (CEU); Tuscan in Italy (TSI); and Mexican ancestry in Los Angeles, California (MEX).

The second dataset (Dataset B) consists in 1092 samples of project 1,000 genomes phase 1 [5], Aboriginal Australian's [12], and our sample's genotype data for 13,177 SNPs. Those SNPs were identified as high quality in the studied sample, genotyped for 90% of the 1000 genomes project samples and the Aboriginal Australian. This dataset included the following individual per population: 89 GBR (British individuals from England and Scotland); 93 FIN (HapMap Finnish individuals from Finland); 100 CHS (Han Chinese South); 55 PUR (Puerto Rican in Puerto Rico); 60 CLM (Colombian in Medellin, Colombia); 14 IBS (Iberian populations in Spain); 85 CEU; 88 YRI; 88 CHB; 89 JPT; 97 LWK; 61 ASW; 66 MXL; and 98 TSI.

To infer the genetic distance, the FST was calculated by Arlequin v.3.5 software [50], and visualized with a discriminant analysis of the principal components (DAPC), included in the adegenet v.1.3-1 library [51] of the R statistical package [52] using dataset A. DAPC was chosen over the conventional principal component analysis (PCA) because it maximizes the differences among groups, which results in a more accurate representation of the differences between the populations. It was also perform the test of “treeness” for 3 populations (*f_3_* statistic [53]) by the Threepop v0.1 software (included in the treemix packge [54]) using dataset B to evaluate the sample admixture. The populations included in this dataset were grouped according to their continent as: Asian (CHB, CHS, and JPT); European (GBR, TSI, IBS, CEU, and FIN); African (ASW, MKK, LWK, and YRI); New Americans (MXL, PUR, and CLM); Australian Aboriginal; and South Native American. The *f_3_* statistic test if a population 1 is admixed regarding the others two populations (this test is formally written as: *f_3_*(X_1_; X_2_, X_3_)), where *f_3_* values significantly negatives indicates that population 1 is admixed.

The admixture analyses were performed using Structure v. 2.3.4 software [55] with 150,000 learning repetitions. The dataset A was analyzed with four to seven subpopulations and dataset B with two to nine subpopulations.

## Supporting Information

Figure S1 **Venn diagram of the shared SNPs among Amerindian sample, CEU, YRI, JPT, and CHB populations.** The Venn diagram represents the total set of SNPs that were found in the Amerindian sample and those SNPs that are shared with the genotyped samples of the CEU (residents of Utah, Nevada, USA with Northern and Western European ancestry from the CEPH collection), YRI (Yoruba in Ibadan, Nigeria), CHB (Han Chinese in Beijing, China), and JPT (Japanese in Tokyo, Japan) populations. Due to the high similarity between the genotypes of the CHB and JPT populations, their representatives were grouped into a single set (CHB+JPT) that represents the mutations that are shared between the Amerindian sample and the CHB and JPT populations.
(EPS)

Figure S2 **Complete population genetic structure analysis of 1,000 genomes project's, Aboriginal Australian and Native South American genotype dataset.** The diagram of genetic contribution was obtained using Structure software for models of 2 to 9 subpopulations. The populations were grouped labeled as follow, according to their major continental ancestry: IND (Native South American individual, this work); ABO (Aboriginal Australian individual, Rasmussen et al. 2011); Eastern Asian (CHS, CHB and JPT); Europeans (CEU, IBS, TSI, FIN and GBR); African (ASW, LWK, MKK and YRI); and New Americans (CLM, PUR and MXL). The plot represents the rate of contribution from each subpopulation (colors) to the samples (x axis).
(TIF)

Table S1 **Total high-confidence SNPs detected.** A complete list of all high-confidence SNPs detected in the sample, including its' chromossomic position relative to hg19 reference genome, the reference and mutant base observed with their relative base coverage and dbSNP identification.
(ZIP)
