# Supplementary figures and images for: High-Throughput Sequencing of a South American Amerindian
Source: PLoS One. 2013 Dec 30;8(12):e83340. doi: 10.1371/journal.pone.0083340 (PMC3875439; doi:10.1371/journal.pone.0083340)

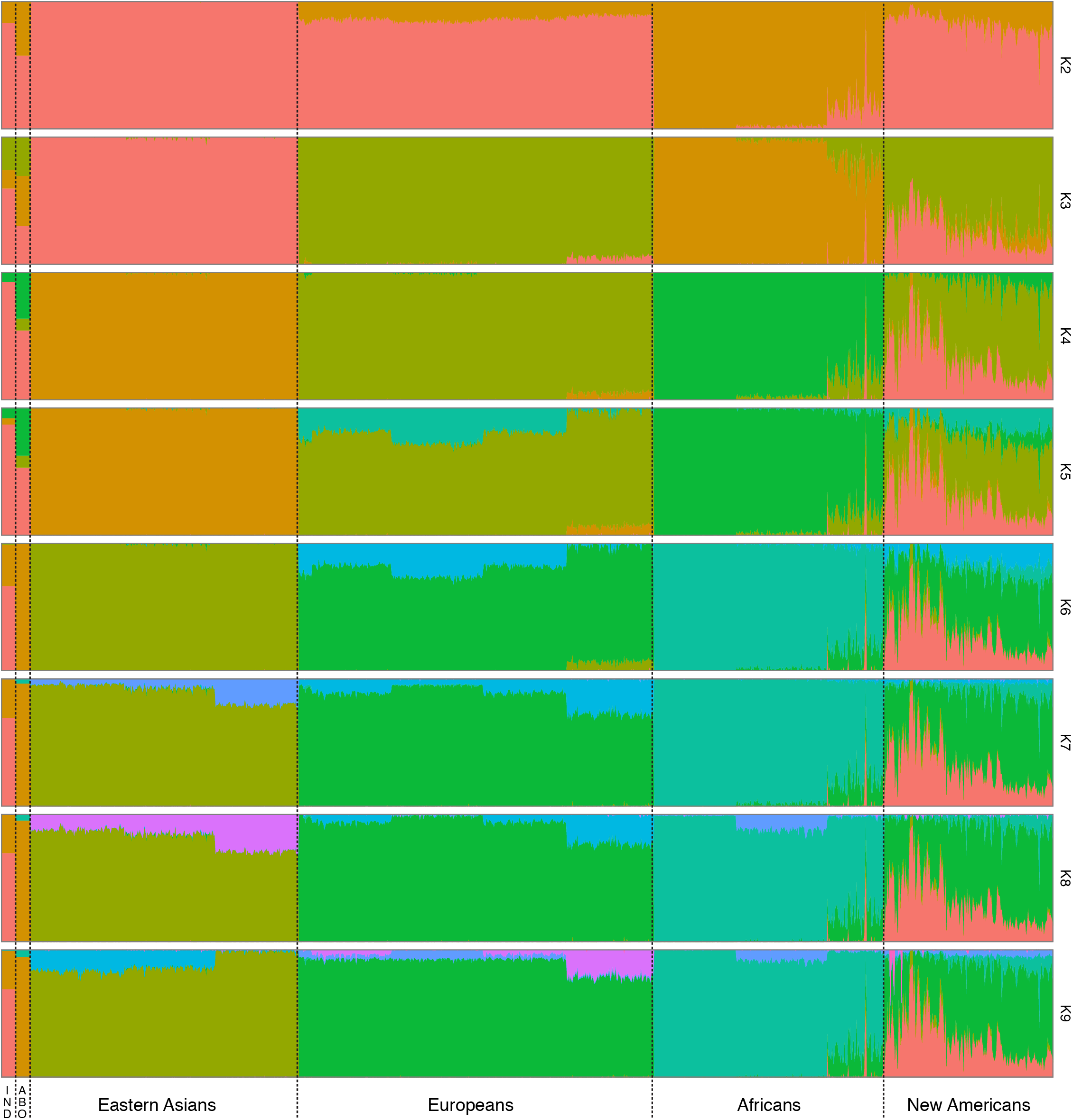

Supplement: Figure S2 — Complete population genetic structure analysis of 1,000 genomes project's, Aboriginal Australian and Native South American genotype dataset. The diagram of genetic contribution was obtained using Structure software for models of 2 to 9 subpopulations. The populations were grouped labeled as follow, according to their major continental ancestry: IND (Native South American individual, this work); ABO (Aboriginal Australian individual, Rasmussen et al. 2011); Eastern Asian (CHS, CHB and JPT); Europeans (CEU, IBS, TSI, FIN and GBR); African (ASW, LWK, MKK and YRI); and New Americans (CLM, PUR and MXL). The plot represents the rate of contribution from each subpopulation (colors) to the samples (x axis). (TIF) [file pone.0083340.s002.tif]
